# Effectiveness of the Inactivated SARS-CoV-2 (Vero Cell) Vaccine in Peruvian Health Workers

**DOI:** 10.3390/life12091318

**Published:** 2022-08-26

**Authors:** Maria Edith Solis-Castro, Alex Jaramillo-Corrales, Rommell Veintimilla Gonzalez Seminario, Noemi Janampa Grados, Idania Edith Mamani Pilco, Karina Elizabeth Vargas Quispe, Lenin Yonel La Torre Rosillo, Mario Neyser Vásquez Dominguez, David Teodoro Enriquez Cusi, Percy Minaya, Karim Jacqueline Pardo Ruiz, Cristian Díaz-Vélez, Vannesa A. Pachas, Ricardo Wesley Alberca, Paul E. Pachas

**Affiliations:** 1 Departamento Académico de Medicina Humana, Facultad de Ciencias de la Salud, Universidad Nacional de Tumbes, Av. Universitaria s/n, Pampa Grande, Tumbes 24001, Peru; 2 Escuela Profesional de Medicina Humana, Facultad de Ciencias de la Salud, Universidad Nacional de San Antonio Abad del Cusco (UNSAAC), Av. de La Cultura 773, Cusco 08000, Peru; 3 Centro Nacional de Epidemiología, Prevención y Control de Enfermedades CDC-Perú, Lima 15072, Peru; 4 Hospital Regional Moquegua, Moquegua 18601, Peru; 5 Red de Salud Bagua, Jr. Ayacucho s/n cdra N° 1, Bagua, Amazonas 01721, Peru; 6 Instituto Nacional Materno Perinatal, Ministerio de Salud, Lima 15001, Peru; 7 Facultad de Medicina, Universidad de Ciencias Aplicadas, Lima 15023, Peru; 8 Ministerio de Salud, Lima 15072, Peru; 9 Instituto de Evaluación de Tecnologías en Salud e Investigación, EsSalud, Lima 14072, Peru; 10 Facultad de Medicina, Universidad Privada Antenor Orrego, Trujillo 13008, Peru; 11 Escuela Profesional de Ingeniería Biomédica, Universidad Nacional Mayor de San Marcos de Lima, Lima 15081, Peru; 12 Laboratorio de Dermatologia e Imunodeficiencias (LIM-56), Departamento de Dermatologia, Faculdade de Medicina FMUSP, Institute de Medicina Tropical da Universidade de Sao Paulo, Sao Paulo 05403-000, Brazil; 13 Instituto Nacional de Salud-Perú, Lima 15072, Peru; 14 Vice Ministerio de Salud Pública, Lima 15072, Peru

**Keywords:** vaccine, effectiveness, SARS-CoV-2, COVID-19

## Abstract

Introduction: The COVID-19 pandemic has caused a global health crisis. Vaccines against this disease have demonstrated variable efficacy and safety, although effectiveness has not been evaluated. In February 2021, the Ministry of Health of Peru approved the emergency use of the inactivated SARS-CoV-2 (Vero Cell) vaccine and initiated vaccination with health personnel at the national level. The objective of the study is to determine the effectiveness of this vaccine to reduce infections, hospitalizations, and deaths due to COVID-19. Methodology: We performed a retrospective cohort study in the period from 23 February to 26 June 2021; data were obtained from the Ministry of Health (including demographic, epidemiologic, clinical, hospital, laboratory results, deaths, and both date and quantity of vaccine doses delivered). The exposed cohort were those who received one or two vaccine doses and the non-exposed were unvaccinated. The events studied were infections, hospitalizations and deaths in the cohorts. We consider a case confirmed for COVID-19 if the test result was positive for SARS-CoV-2, via PCR or antigen test. Effectiveness was measured with incidence density ratio and risk. Confounding factors were controlled using a Poisson model with robust variance. Results: We enlisted 520,733 health workers, of whom 415,212 had two vaccine doses and 105,521 were unvaccinated. The median age was 40 years (IQR: 32–50), and 65.6% were female. The effectiveness of two vaccine doses fourteen days after application adjusted by age, sex, hospitalization, and antecedent of having the infection was 90.9% (95% CI: 85.5–94.2%); effectiveness to avoid death from COVID-19; 67.7% (60.1–73.8%) effectiveness to avoid hospitalizations; and 26.3% (23.8–28.6%) effectiveness to reduce the risk of infection by SARS-CoV-2 relative to the unvaccinated cohort. Conclusions: The inactivated SARS-CoV-2 (Vero Cell) vaccine used in two doses has an acceptable effectiveness against death and risk of hospitalization, whereas it has less effectiveness in preventing COVID-19 infection.

## 1. Introduction

The COVID-19 disease pandemic caused by the severe acute respiratory syndrome coronavirus 2 (SARS-CoV-2) has caused a global health crisis [1], with high social and economic costs [2]. The COVID-19 mortality rate is higher among older persons, those with comorbidities and health workers, such as doctors, nurses and technical personnel, who are up to three times more likely to be infected compared to the general population, likely due to greater disease exposure within the healthcare sector [3].

From the first months of the pandemic, the development of vaccines against SARS-CoV-2 began, with acceptable efficacy and safety, and large-scale production and distribution worldwide [4]. The Vaccine Center at the London School of Hygiene & Tropical Medicine, through its COVID-19 vaccine tracker, registers 322 vaccine candidates developed against SARS-CoV-2; of these, 225 are in the pre-clinical phase, 97 in the clinical phase, and 17 have been approved for emergency use in multiple countries [5].

Phase III clinical trials have demonstrated that several vaccine candidates are safe and effective with acceptable immunogenicity, including BNT162, mRNA-1273, ChAdOx1 nCoV-19 (formerly AZD1222), BBIBP-CorV and Ad26.COV2.S. Consequently, numerous countries have started vaccination against COVID-19 [6]. Nonetheless, despite the efficacy demonstrated in phase III clinical trials, little is known about the vaccine effectiveness under real world conditions, [7,8] especially with the appearance of the variants of interest and concern with an increased ability to evade the immune response [9], creating further uncertainty.

In August 2020, Peru created the “Multisectoral Commission of temporary nature in charge of monitoring actions aimed at the development, production, procurement, donation and distribution of vaccines and/or treatments against COVID-19”. During its tenure, the Commission formulated recommendations for the procurement of vaccines from Pfizer, Sinopharm, AstraZeneca, and Johnson & Johnson, as well as the COVAX Facility for vaccine administration.

In January 2021, Peru′s Ministry of Health granted an exceptional authorization for the importation and use of the inactivated SARS-CoV-2 (Vero Cell) vaccine from the manufacturer Beijing Institute of Biologial Products Co., Ltd. (BIBP, Beijing, China)–China [10]. The implementation of the vaccine began on 9 February 2021, among healthcare workers in both the public and private sectors [11]. The phase I and phase II trials of the BBIBP-CorV vaccine demonstrated the vaccine to be both safe and well tolerated, with a humoral response against SARS-CoV-2 in all recipients of the vaccine on day 42 [12]; hence, its implementation was recommended on days 0 and 21–28 [13]. The phase III study undertaken in the United Arab Emirates showed an efficacy of 79.34% (preliminary results) [14].

Vaccination against COVID-19 in Peru began [15] with the increase in cases during the second wave of COVID-19 infections, with an attack rate of 4.8% in the general population [16]. Among medical doctors, there was an attack rate of 14.3% and a 3.5% fatality rate [14], the third highest rate in Latin America for doctors infected and killed by COVID- 19. To face this situation, the Ministry of Health of Peru approved the National Vaccination Plan and considered the healthcare workforce in Phase I [16]. The objective of the study is to determine the effectiveness of the inactivated SARS-CoV-2 (Vero Cell) vaccine in reducing infection, the severe forms of the disease that cause hospitalization, and death in health workers in Peru.

## 2. Materials and Methods

### 2.1. Study Design and Data Sources

A retrospective cohort study was conducted in health workers from 9 February to 26 June 2021, using secondary information from databases of the Ministry of Health that included demographic, epidemiological, clinical, hospital data, data from the laboratory (polymerase chain reaction test (PCR) and antigenic tests for the detection of SARS-CoV-2), death, as well as both the date and quantity of vaccination doses against SARS-CoV-2.

The integrated database was built from the amalgamation of the following data sources: the national vaccination registry of the General Office of Information Technology; the National Registry of Health Personnel; Smart Health System (ESSI) records of the Social Health Insurance of Peru; records of the Comprehensive Health Insurance (SIS); records from the Integrated System for COVID-19; clinical and laboratory data for SARS-CoV-2 from the laboratory information system (NetLab v2.0) of the National Institute of Health (NIH); and the information system of the epidemiological surveillance of COVID-19 of the National Center of Epidemiology, Prevention and Control of Diseases of Peru. The information on COVID 19-related deaths was obtained from the National Informatic System of Defunctions of the General Office of Information Technology. The type of profession was identified from the database of eleven professional associations in Peru.

The General Office of Information Technology of the Ministry of Health joined the different databases, providing the researchers with identification data of the anonymized subjects and the study’s variables of interest. The quality control of the data was performed by the institution responsible for the database, the General Office of Information Technology, and the researchers.

### 2.2. Study Population

The study participants were health workers of both sexes, over 18 years of age, and comprised of both health professionals and technicians who deliver healthcare services as well as administrative and support services (including security, maintenance, etc.) across Peru’s 24 departments and the Constitutional Province of Callao, in the three natural regions, at all the three levels (I, II and III) of the public and private health systems, providing services ranging from outpatient care to hospitalization and critical care for severe cases of COVID-19 [15].

The eligibility criteria for study inclusion were whether the health worker was vaccinated with the inactivated SARS-CoV-2 (Vero Cell) vaccine and registered in the database of vaccinated persons of the Ministry of Health of Peru. Workers who presented a severe event supposedly attributable to a second dose of vaccination administered after 25 May were excluded from the analysis; patients were also excluded if they presented one of the events of interest between the date of their first dose, and 14 days after their second vaccine dose. In the unvaccinated cohort, subjects were excluded who presented one of the events of interest from 9 February 2021 and 22 February 2021 (14 days from the start of vaccine deployment).

The retrospective monitoring for the two cohorts ran from 25 June 2021 to 14 days after the administration of the second dose for the vaccinated cohort, and up to 14 days after the start of the vaccination of health personnel (23 February) for the unvaccinated cohort. The monitoring period ended if the event of interest (infection, hospitalization or death) presented in both cohorts. The monitoring days were estimated through the difference between the day of the end of the study (26 June) or the presentation of the event of interest, and 14 days after the application of the 2nd dose, for the vaccinated cohort, or February 23 for the unvaccinated cohort.

In the vaccinated cohort, we considered infected cases if they had a positive PCR or antigenic test result 14 days after the 2nd dose was administered. In the unvaccinated cohort, we considered infected cases if they had a positive PCR or antigen result since 23 February 2021 (two weeks after starting the application of vaccines in Peru) to guarantee the monitoring of both cohorts throughout the same period of the pandemic. We considered an antecedent of previous infection to SARS-CoV-2 if the participant had a positive PCR, antigenic or serological test since the beginning of the pandemic, from March 2020 to 9 February 2021.

The PCR tests were performed in either the laboratories of the NIH, or in public or private laboratories accredited by the NIH. The antigenic tests were performed in each health establishment or in the house of the health worker as part of the epidemiological surveillance system. Samples for laboratory tests were collected in case the health worker had symptoms, or periodically as part of the regular surveillance of COVID-19 in health workers.

The inactivated vaccine (Vero Cell) from the manufacturer BEIJING INSTITUTE OF BIOLOGIAL PRODUCTS (BBIBP-CorV against SARS-CoV-2) was applied according to the suggestions in the laboratory insert, the first dose on day 0 and the second dose 21 days later.

### 2.3. Criteria for Analysis

We considered the following events for the study: infection, hospitalization and death related to the SARS-CoV-2 virus. Any subject participant with at least one positive test result was defined as infected. Subjects with a registered hospital admission date to a health facility and at least one recorded, positive test result were defined as hospitalized. Any subject registered in the National System of Deaths of Peru (SINADEF); Epidemiological Surveillance Notification System (NOTISP); Comprehensive Health Insurance (SIS); or Smart Health Service (EsSI) with a diagnosis related to COVID-19 and with a positive test result were defined as having died from COVID 19. The tests considered as a confirmatory diagnosis of SARS-CoV-2 in our study were PCR or antigenic tests.

### 2.4. Statistical Analysis

We used the statistical program STATA v16 (Serial number: 501606349486, StataCorp LLC, College Station, TX, USA). We carried out a descriptive analysis of the sociodemographic variables of the study population, including age, gender, professional association, occupational group and department of residence. Continuous variables were summarized as means and medians with standard deviation or interquartile ranges according to the Gaussian distribution. Categorical variables were summarized with frequencies and percentages.

We used the incidence density ratio as the measurement of events; this is the quotient between the total number of cases of a given event (infected, hospitalized or deceased) and the person-days of follow-up of each participant during the study period, multiplied by 1000.

Vaccine effectiveness was defined as the reduction in the incidence density ratio expressed in percentages. We considered the events presented 14 days after the second dose in the vaccinated cohort and 14 days after the start of vaccination in health workers for the cohort that did not receive any dose of vaccine, using the following formula:

Effectiveness (VE) = (1 − Incident density ratio) × 100

We used a Poisson regression model with robust variance to adjust the results of the incidence ratio for risk factors or confounding variables such as age, sex, previous history of infection, hospitalization, and death. The 95% confidence intervals were estimated for each measure [17].

## 3. Results

**Figure 1:**
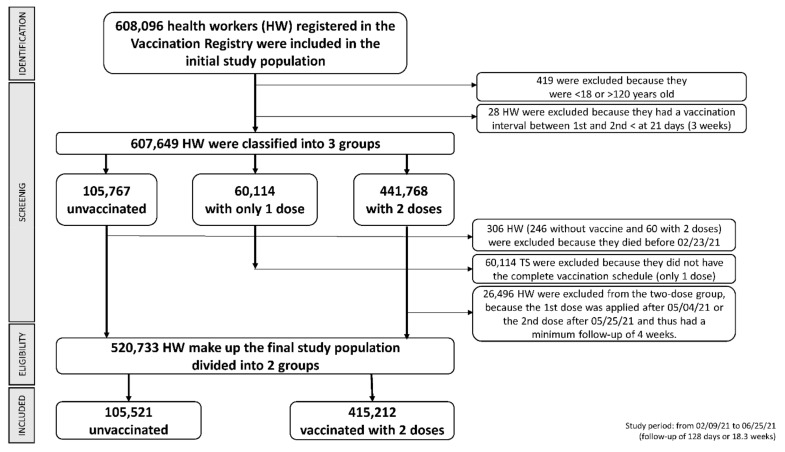
Study participants and eligibility.

We enrolled 520,733 health workers, of which 415,212 received two vaccine doses and 105,521 did not receive any dose; we excluded 60,114 health workers who were vaccinated with a single dose (Figure 1).

The median age of the subjects was 40.0 years old (IQR: 31–51), and 65.6% were female. The median time interval between the administration of the first and second doses of the SARS-CoV-2 vaccine was 21 days (IQR: 21–22).

Table 1 shows the main characteristics of both the vaccinated and unvaccinated cohorts.

### 3.1. Vaccine Effectiveness in Preventing SARS-CoV-2 Infection

A total of 16,864 cases of SARS-CoV-2 were confirmed with PCR or antigenic tests. Of these, 10,560 received two doses of vaccine and 6304 received none.

The crude incidence density ratio for infection is 0.632 (95% CI 0.61–0.65); adjusted for age group (≥60 or <60 years), gender, previous history of infection, hospitalization and death, this is 0.737 (95% CI 0.714–0.762). The effectiveness of the vaccine in reducing SARS-CoV-2 infections is 26.3% (95% CI 23.8–28.6%) (Table 2).

### 3.2. Vaccine Effectiveness in Hospitalized for SARS-CoV-2

A total of 513 subjects were hospitalized for SARS-CoV-2, of which 170 had two doses of the vaccine and 343 received none. The incidence density ratio for hospitalization is 0.189 (95% CI 0.16–0.24), while the adjusted incidence density is 0.323 (95% CI 0.262–0.399). The effectiveness of two doses of vaccine in avoiding hospitalization for SARS-CoV-2 is 67.7% (95% CI 60.1–73.8%).

### 3.3. Vaccine Effectiveness in Deaths from SARS-Co-V-2

A total of 234 deaths from SARS-CoV-2 were identified, of which 29 had two doses of the vaccine and 205 were not vaccinated. The density ratio for the incidence of deaths is 0.054 (95% CI 0.034–0.075); the adjusted incidence density ratio is 0.092 (95% CI 0.058–0.145). The effectiveness of the vaccine to prevent deaths in the two-dose vaccine is 90.9% (95% CI 85.5–94.2%).

**Figure 2:**
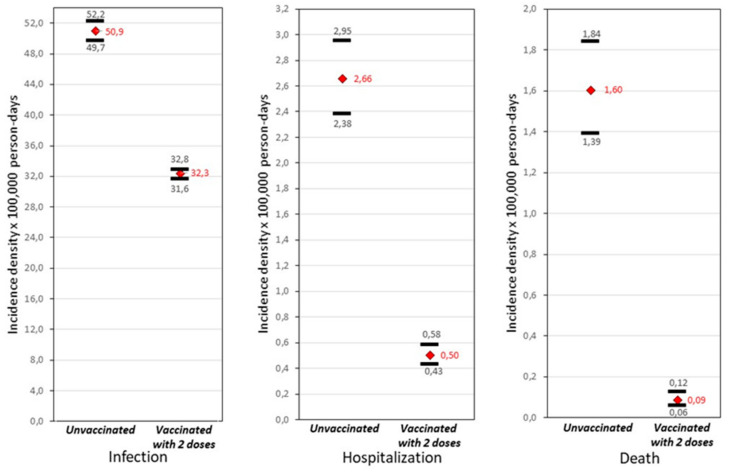
Crude incidence density rates for each event (CI 95%) in health workers, Peru Feb.–Jun. 2021.

It was found that there is a difference in incidence density x per 100,000 person-days between the two cohorts of 1.6-fold for infection, 5.3-fold for hospitalization, and 17.8-fold for death. The risk reduction effect between those vaccinated with two doses and those not vaccinated is notable, with infection being the event with the highest probability of occurrence in both groups (see Figure 2).

**Figure 3:**
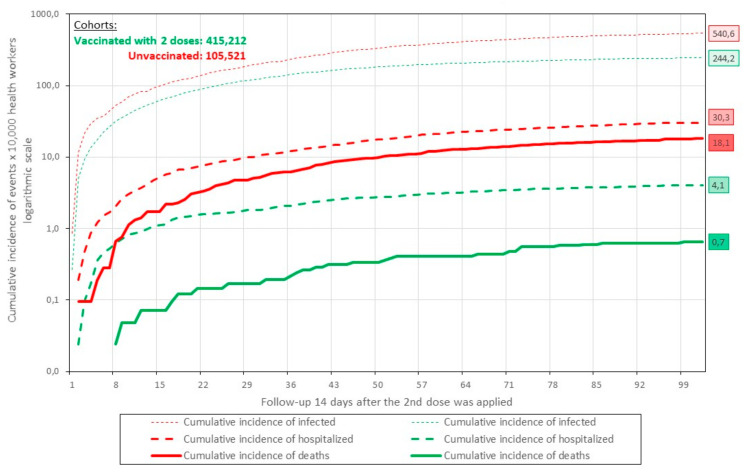
Follow-up and ridk of infection, hospitalization and death from COVID-19 per 10,000 vaccinated (inactivated SARS-CoV-2, Vero Cell) and unvaccinated health workers in Peru February–June 2021.

In the follow-up of both cohorts for 15 weeks (Figure 3), it can be seen that the effect of the vaccine to prevent the events under study was considerable and sustained throughout the process. Thus, the risks decreased from 540.6 to 244.2 for infection, from 30.3 to 18.1 for hospitalization, and from 4.1 to 0.7 per 10,000 health workers for death. However, as the weeks go by, it seems that the protection decreases. A longer follow-up time is needed to determine the best time to receive a booster.

**Figure 4:**
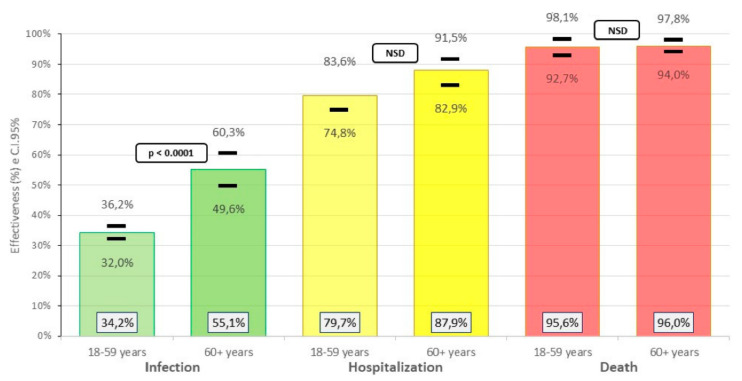
Effectiveness of the inactivated SARS-CoV-2 (Vero Cell) vaccine against COVID-19 according to age, in Peruvian health workers, February–June 2021.

The greater effectiveness of the vaccine in the group of 60 years or older was for infection events (55.1% vs. 34.2% (*p* < 0.001)) and hospitalization (87.9% vs. 79.7%), compared to the group age from 18 to 59 years, while similar data were found for deaths (96.0% vs. 95.6%) (Figure 4).

**Figure 5:**
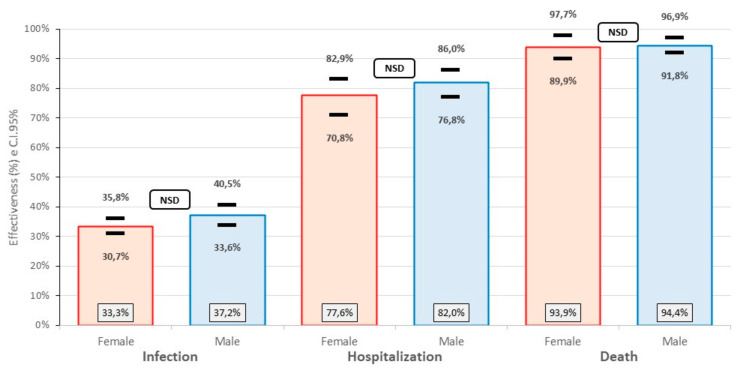
Effectiveness of the inactivated SARS-CoV-2 (Vero Cell) vaccine against SARS-CoV-2 according to sex, in Peruvian health workers, February–June 2021.

When comparing the effectiveness of the vaccine to prevent the three events according to sex, no significant differences were found in any of them (infection, hospitalization and death). Therefore, the effectiveness is the same in women and men. These effectiveness data confirm what was analyzed in a systematic review and meta-analysis that concludes that, despite the significant biological and behavioral differences between both sexes, no significant differences were found in the efficacy of the COVID-19 vaccines (Figure 5).

**Figure 6:**
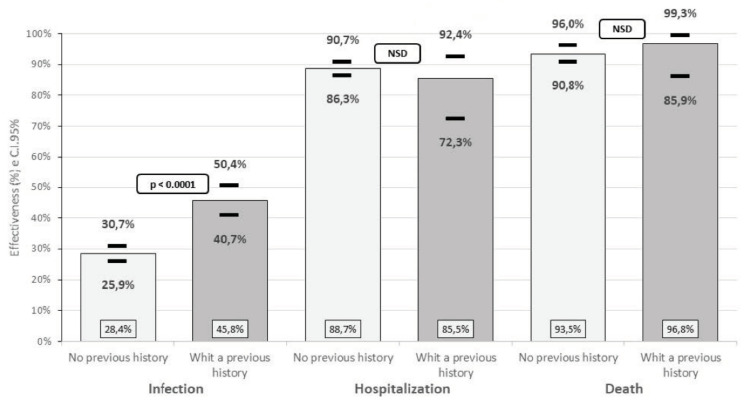
Effectiveness of the inactivated SARS-CoV-2 (Vero Cell) vaccine against COVID-19 according to previous history of infection, in Peruvian workers, February–June 2021.

A previous history of infection significantly increases the effectiveness of the vaccine against a new infection, increasing from 28.4% to 45.8% (*p* < 0.0001). A previous history of infection decreases the effectiveness against hospitalization, while it increases the effectiveness against death, but these variations are not significant (Figure 6).

**Figure 7:**
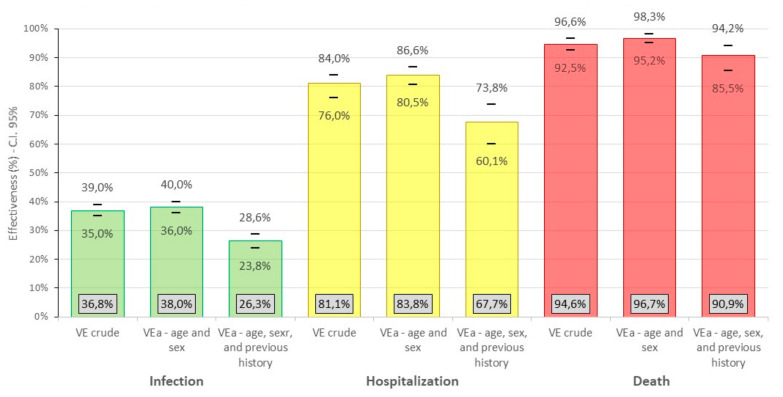
Crude and adjusted effectiveness of the inactivated SARS-CoV-2 (Vero Cell) vaccine against COVID-19, in Peruvian health workers, February–June 2021.

In Figure 7, it can be seen that the effectiveness of the vaccine against infection decreases when adjusted for age, sex and previous history against the crude rate and when adjusted only for age and sex. We observed a similar situation with adjusted effectiveness against hospitalization; however, in the effectiveness of the vaccine against death, the rate adjusted for age, sex and previous history of infection decreases the effectiveness when compared with the rate adjusted for age and sex, but not when compared to the crude rate.

## 4. Discussion

The effectiveness of the inactivated SARS-CoV-2 (Vero Cell) vaccine applied in two doses to health workers is 90.9% to prevent death, 67.7% to prevent hospitalization and 26.3% to reduce the risk of infection by SARS-CoV-2. This is the first large cohort study on the inactivated SARS-CoV-2 (Vero Cell) vaccine effectiveness.

For the randomized phase 3 clinical trial on the effect of two inactivated vaccines against SARS-CoV-2 on symptomatic COVID-19 infection in adults, with an average age of 36.1 years, 84.4% of them men, where 94.6% received two doses, and an average monitoring of 77 days, the preliminary results reported a vaccine efficacy compared to placebo, of 72.8% (95% CI: 58.1–82.4%) for WIV04 and of 78.1% (95% CI: 64.8–86.3%) for HBO2. Two severe cases of COVID-19 occurred in the placebo-only group and none in the vaccine groups [18]. These results differ from this study, in the analysis period of 21 and 28 days recommended by the manufacturer, since this analysis also included people vaccinated with a second dose administered 28 days after the initial dose; this could alter the protective effect of the vaccine. In the same way, the study′s monitoring period is up to 100 days following administration of the second vaccine dose. There were more than 500 thousand people included in the study, who, due to the work they perform, constitute a group with high exposure to the virus, representing the effectiveness of the study with the largest population followed.

On 7 May 2021, the Strategic Advisory Group of Experts on Immunization of the WHO, based on the evidence available, recommended the BBIBP-CorV vaccine for adults over 18 years of age over a two-dose schedule. With an interval of three to four weeks between doses, the reported efficacy to prevent symptomatic and hospitalized disease is 79% [19].

Worldwide, there are studies with similar results, while others have different methodology and findings. A study conducted in Israel, which included all vaccinated and unvaccinated people nationally, measured events (illness, infection, hospitalization, and death) at 14 and 20 days after the first dose, as well as 7 days after the second dose, between 20 December 2020 and 1 February 2021. For documented infection, the Israel study reported a 46% effectiveness following the first dose (95% CI, 40–51) and 92% following the second (95% CI, 88–95), respectively. Likewise, the study reported for symptomatic COVID-19: 57% (95% CI, 50 to 63) and 94% (95% CI, 87 to 98) for hospitalizations; 74% (95% CI, 56 to 86) and 87% (95% CI, 55 to 100) for severe disease; 62% (95% CI, 39 to 80) and 92% (95% CI, 75 to 100)to prevent death; and 72% (95% CI, 19 to 100) during days 14 to 20 after the first dose [20]. These results differ from those found in the present, probably because the study included the entire national population, with a different vaccine, and was also matched by clinical and demographic characteristics.

Compared with other vaccine types, the difference in effectiveness could be greater. A study conducted in Israel with the BNT162b2 vaccine (Pfizer Inc., New York, NY, USA), which used information from the national surveillance system corresponding to the first 4 months of the national vaccination campaign, reported that the effectiveness in reducing infection from the seventh day of the second dose was 95.3% (95% CI 94.9–95.7); 91.5% (95% CI 90.7–92.2) for asymptomatic infection; 97.0% (96.7% CI 96.7–97.2) for symptomatic infection; 97.2% (CI 95% 96.8–97.5) for hospitalization; 97.5% (95% CI 97.1–97.8) for severe COVID-19; and 96.7% (95% CI 96.0–97.3) for death from COVID-19 [21].

Another study conducted in Israel using the same BNT162b2 vaccine reported a 51.4% vaccine effectiveness against SARS-CoV-2 infection at 13–24 days after immunization with the first dose. These findings were similar in adults aged 60 years and over (44.5%); in those under 60 years of age (50.2%); men (52.1%); and women (50.0%) [22]. The reported values are also superior to the effectiveness found at present for the BBIBP-CorV vaccine.

On the other hand, a cohort study conducted in England with the BNT162b2 vaccine, also among health personnel, reported an incidence density of 14 infections per 10,000 person-days in the unvaccinated cohort; eight infections per 10,000 person-days at 21 days after the first dose; and four infections per 10,000 person-days 7 days after the second dose in the vaccinated cohort. In this way, it was determined that a single dose of the BNT162b2 vaccine was 70% (95% CI 55–85) effective against infection twenty-one days after the first dose, and 85% (95% CI 74–96) effective seven days after the second dose in the study population [23].

However, the BNT162b2 vaccine, when evaluated in effectiveness studies, showed different results. After the second dose, between weeks 1 and 2, the effectiveness in reducing positive cases was 73% and 85%, respectively. After 14 days of the second dose, the effectiveness was 89% to prevent hospitalizations and 97% to prevent severe disease [13].

If we consider other vaccines types such as Ad26.COV2.S (Janssen/Johnson & Johnson, New Brunswick, NJ, USA), which uses a viral vector, the protection may also be greater than that offered by BBIBP-CorV. In a phase III efficacy trial, the single-dose Ad26.COV2.S vaccine was 66.9% effective (95% CI 59.0–73.4) in preventing moderate to severe COVID-19 infections starting 14 days after vaccination. Efficacy was higher at 78% and 85%, 14- and 28-days following vaccination, respectively [24].

On the other hand, the ChAdOx1 nCoV-19/AZD1222 vaccine (University of Oxford, AstraZeneca, and the Serum Institute of India), in an interim report of a phase III study, showed an efficacy of 70.4% (95% CI 54.8–80.6) to prevent symptomatic COVID-19 at 14 days or more following the second dose. In this same study, a subgroup of participants was inadvertently given a lower dose of vaccine for the first of the two doses. In this subgroup, the efficacy reached 90.0% (95% CI 67.4 to 90.0), much higher than the 62.1% (95% CI 41.0 to 75.7) among those who received the full dose. Although the reasons for this difference are uncertain, the CI values suggest the difference in efficacy may not be statistically significant [25].

In a later report from this same study, the efficacy of the ChAdOx1 nCoV-19/AZD1222 vaccine for symptomatic COVID-19 was 76%, 21 days after receiving the first dose until receiving the second dose, or 90 days following the first dose, suggesting protection with a single dose [26]. Another report based on preliminary results from a placebo-controlled trial conducted in the United States, Chile, and Peru reported similar findings at two full doses four weeks apart. The reported values were 76% efficacy against symptomatic COVID-19 15 days following the second dose, 100% against serious or critical illnesses and hospitalization, and 85% efficacy for symptomatic COVID-19 in people aged 65 and older [27].

Even though these data mostly correspond to other types, and the methodology used is different (efficacy vs. effectiveness), the findings suggest less protection of the inactivated SARS-CoV-2 (Vero Cell) vaccine against SARS-CoV-2 to reduce infection, hospitalization and death associated with COVID-19, 14 days after the second dose. However, they are still acceptable, meeting the World Health Organization′s (WHO) vaccine standard.

Similarly, our study results differ from the BBIBP-CorV vaccine′s preliminary efficacy data for the prevention of symptomatic SARS-CoV-2 infections (78.1%), even with the post hoc results from the multicentered randomized clinical trial, which included asymptomatic cases (73.5%) and was later approved by the WHO for the use and marketing of the BBIBP-CorV vaccine [18,28].

These study results may differ because of the criteria for evaluating the effectiveness of the vaccine. The multicentered study evaluated effectiveness among symptomatic infections, while our study included both symptomatic and asymptomatic infections. Compliance with the definition of a suspected case of the standard epidemiological surveillance could not be 100% controlled. Despite the non-compliance at the hospital level of the periodic screenings of health personnel and the greater accessibility to diagnostic tests in case of work contacts, the suspected case definition generated an increase in the number of positive cases detected, underestimating the real effectiveness the vaccine may have against symptomatic disease.

Furthermore, the risk of exposure of health workers is greater, in terms of exposure time, viral load and in the likely exposure to multiple COVID-19 variants while delivering patient care, driving a decrease in vaccine effectiveness estimates.

Evaluating these results for public health in terms of infection and transmission is more challenging than for the results of the disease. Therefore, future research will include, in addition to the separate analysis of the effectiveness among symptomatic and asymptomatic patients, viral quantification, active surveillance in the professional and domestic environments and patient motivation for testing, whether due to COVID-19 exposure or routine practice, to refine estimates for vaccine effectiveness for disease infection and transmission.

Several studies of the early effectiveness of the vaccine against SARS-CoV-2 in medical settings for health workers have shown that vaccination in health personnel has a positive effect on the preservation of the workforce, through a marked reduction in disease incidence in medical personnel [29,30,31], including asymptomatic infections [32]. These studies rely on policies of weekly testing to identify asymptomatic infections, thus, facilitating the detection of asymptomatic SARS-CoV-2 infections after vaccination. In contrast, our analysis relies on regulations that only establish the testing of symptomatic personnel, overlooking asymptomatic infections. Such results are consistent with what we have observed, in such a way that, regardless of the brand or type of vaccine, immunizations have proven to be the most cost-effective public health intervention. It is highlighted that, despite this, the need to continue with complimentary interventions of social distancing, epidemiological surveillance, and laboratory and clinical monitoring, is emphasized. This is a crucial aspect that Peru must consider as new COVID-19 variants circulate and given the still limited knowledge of the effectiveness existing vaccines would have on them.

Another study has raised the need to evaluate vaccine effectiveness through the measurement of the antibody levels to determine the immune response to COVID-19 vaccination [33]. Although vaccines may provide long-term immunity via T cells and memory B cells [34], neutralizing antibody production after vaccination provide an immunological biomarker correlated with protection against SARS-CoV-2 in humans. Therefore, anti-SARS-CoV-2 antibodies are a valuable tool to assess the protective immunity after vaccination, and to aid in determining immunization schedules.

Additionally, our findings show that although the vaccine has a protective effect, this effect decreases five weeks after administration of the second dose for all groups observed (infections, hospitalizations, and deaths from COVID-19). Studies performed with mRNA vaccines indicate that the onset of protection was observed as early as 12 days after the administration of a single vaccine dose. The adaptive immune response that coincides with this onset of protection could represent the necessary elements of immunity against COVID-19 [35]. Preliminary data identified a reduced T and B cells response to inactivated vaccine in the elderly [36], suggesting the need to apply a third dose of an alternative vaccine, following the most common recommendation with mRNA vaccines.

The sensitivity of the diagnostic test used (65% for PCR tests and 56.2% for antigen tests [37] on average), directly influences the study results. This is due to the frequency of “false negative” test results. Additionally, the 53 different lineages of the SARS-CoV-2 virus identified to date also influence study results; this include variants identified in Lima, such as the B.1.1.7 (Alpha) lineage—the British variant, and the P1 (Gamma) lineage—the Manaus–Brazil variant (also identified in Huánuco and Loreto), which constituted 39.9% of the COVID-19 cases last sampled in Lima [38]. These variants increase viral transmission, the severity of COVID-19 symptoms, mortality rates, and importantly, they can also decrease the effectiveness of diagnostic methods, influencing the effectiveness of available vaccines [39,40].

Reports from Israel have linked vaccination with an increase in COVID-19 cases shortly following vaccination, driven by an increase in community and healthcare-related exposures. These same reports note that the coexistence of vaccination with the rapid spread of COVID-19 is a period in which vaccination-related symptoms pose a diagnostic dilemma, convoluting a vaccination-driven adverse reaction with a new COVID-19 infection. Considering these results, the COVID-19 cases identified in our study require additional monitoring and research to determine whether symptom onset began before vaccination. Likewise, the authors of another publication related to the same study conclude that both vaccinated subjects and unvaccinated controls must be sampled in order to determine a true decrease in infections, especially in asymptomatic cases among vaccinated persons [41].

Therefore, a second vaccine and the resulting benefit of immunization against SARS-CoV-2 with full vaccination should be emphasized.

The COVID-19 variants are emerging problems that may affect the effectiveness of existing vaccines. For this reason, the NIH has communicated the findings of genomic sequencing in Peru [42]. The NIH reports that, of 1714 samples extracted from March 2020 to May 2021, 71.6% (1228/1714) belong to the lambda or C.37 variant, which would be proportionally dominant at the time of evaluating the effectiveness of the BBIP-CorV vaccine. The same report shows that 14.1% (242/1714) of the sequenced samples correspond to the gamma or P1 variant (the “Brazilian variant”), present in 22 regions of Peru, except for Puno and Ancash. In the period of the study, neither the efficacy nor the effectiveness of the inactivated SARS-CoV-2 (Vero Cell) vaccine on the new SARS variants could be determined. CoV-2 (Delta and Omicron) appeared later.

To determine the effectiveness of the inactivated CoronaVac vaccine, a case-control study was conducted among people aged 70 years and over in Sao Paolo, Brazil. It was conducted in relation to the P1 variant (which represented 85% of the genotyped samples in the analysis period). Using surveillance and vaccination records, the case-control study found two doses of the CoronaVac vaccine to have an adjusted effectiveness of 41.6% (95% CI: 26.9 to 53.3) and 18.2% (95% CI 0.0 to 33.2) in the period ≥14 days and 0–13 days, respectively. When analyzed by age, the vaccine effectiveness decreases significantly after 14 days of the second dose, falling from 61.8% in the 70- to 74-year-old group to 28.0% in the over-80 year old group. These estimates approximate those identified in our study. [43].

This raises four major concerns, including: effects on viral transmissibility, the severity of the disease, the reinfection rates or escape from natural immunity, and the efficacy of the vaccine or escape from vaccine-induced immunity [44]. The evaluation of the effectiveness of vaccines on new variants could generate biases and confounding factors related to the variable risk of infection over time and the probability of receiving the vaccine or not [45].

The neutralizing activity of the serum for the B.1.351 (or the Beta, South African) variant among vaccinated people was lower by a factor of 1.6 to 8.6 for the BBIBP-CorV vaccine (Sinopharm), the BNT162b2 vaccine (BioNTech-Pfizer) and the mRNA-1273 (Modern) vaccine. It was also lower by a factor of up to 86, including complete immune escape, for the AZD1222 (Oxford-AstraZeneca) vaccine. This is why the administration of the latter vaccine was interrupted. The neutralizing activity for the P.1 variant among vaccinated persons was lower by a factor of 6.7 for the BNT162b2 vaccine and by a factor of 4.5 for the mRNA-1273 vaccine [44,46].

What has been described raises the hypothesis that the inactivated SARS-CoV-2 (Vero Cell) vaccine might be less effective due to mutations in the SARS-CoV-2 virus, specifically the predominant C.37 variant in the current epidemiological scenario. This should be corroborated with additional research. In the meantime, however, it puts Peru′s health system on alert.

The strength of our study was the access to official information from the Ministry of Health, where the data were collected from the databases that served as a source of information for the study, which facilitated the capture and recapture of the data.

Despite the limitations of using national data for this analysis, it is necessary to incorporate hospitalization data from other parts of Peru′s health system, including from the private sector, the armed forces, and the police force, for data integrity and quality control, in order to prevent omission in the registry of study information sources.

Further analysis should also include other relevant variables, such as exposure difference to the SARS-CoV-2 virus by types of activity carried out in workplaces (service provision, administrative tasks, etc.). Future analysis should also aim to complete the individual clinical information of infected, hospitalized, and deceased cases. Additionally, as it is an analysis of secondary sources, there may be information biases.

## 5. Conclusions

In conclusion, two doses of the inactivated SARS-CoV-2 (Vero Cell) vaccine applied to health workers from MINSA-Peru has acceptable effectiveness in reducing hospitalizations and death from COVID-19 fourteen days after the application of the second dose. The effectiveness in reducing SARS-CoV2 infection, however, is low.

It is necessary to continue surveillance studies for longer periods and to include other variables which were not included in the present analysis. Additionally, pre-vaccination periods should be compared and the effect of vaccines against the COVID-19 variants identified in Peru should be evaluated, as well as the need for additional vaccine doses.

## Figures and Tables

**Table 1:** Characteristics of the study cohort.

Variable	Unvaccinated		Cohort Vaccinated		Total	
	Cohort		with 2 Doses			
	n_0_	%	n_2_	%	N	%
**Gender**						
Female	72,746	68.9%	268,855	64.8%	341,601	65.6%
Male	32,775	31.1%	146,357	35.2%	179,132	34.4%
**Median age (range)**	37	(31–46) *	40	(32–51) *	40	(31–51) *
**Mean age (SD)**	39.75	11.89	42.66	12.98	42.04	12.79
**Age group**						
18–29	20,771	19.7%	65,914	15.9%	86,685	16.6%
30–59	77,569	73.5%	297,624	71.7%	375,193	72.1%
60 or more	7181	6.8%	51,674	12.4%	58,855	11.3%
**Previous history of infection**						
Present	19,802	18.8%	130,889	31.5%	150,691	28.9%
Absent	85,719	81.2%	284,323	68.5%	370,042	71.1%
**Population**	**105,521**	100%	**415,212**	100%	**520,733**	100%

* IQR: Updated 26 June 2021.

**Table 2:** Incidence density ratio and effectiveness of the inactivated SARS-CoV-2 (Vero Cell) vaccine against COVID-19, according to gender, age and previous history *.

Categories, Outcomes, and Vaccination Status	Events	Persons-Days of Follow-Up	Incidence Density per 1000 Days of Follow-Up	Crude Incidence Density Ratio (CI 95%)	z	*p*-Value	Effectiveness of the 2 Doses of the Vaccine (CI 95%) **
**Gender**							
**Female**							
Infection							
*Unvaccinated*	4263	8,684,163	0.49089	0.667	−20.78	<0.0001	**33.3%**
*Vaccinated with 2 doses*	6975	21,290,825	0.32761	(0.64–0.69)			(30.7–35.8)
Hospitalization							
*Unvaccinated*	156	8,390,243	0.01859	0.224	−14.56	<0.0001	**77.6%**
*Vaccinated with 2 doses*	81	19,483,688	0.00416	(0.16–0.24)			(70.8–82.9)
Death							
*Unvaccinated*	68	8,972,580	0.00758	0.061	−8.08	<0.0001	**93.9%**
*Vaccinated with 2 doses*	10	21,657,884	0.00046	(0.034–0.075)			(89.9–97.7)
**Male**							
Infection							
*Unvaccinated*	2041	4,109,548	0.49665	0.628	−16.73	<0.0001	**37.2%**
*Vaccinated with 2 doses*	3585	11,492,768	0.31194	(0.60–0.66)			(33.6–40.5)
Hospitalization							
*Unvaccinated*	187	3,972,196	0.04708	0.180	−13.30	<0.0001	**82.0%**
*Vaccinated with 2 doses*	89	10,486,394	0.00849	(0.14–0.23)			(76.8–86.0)
Death							
*Unvaccinated*	137	4,241,419	0.03230	0.056	−12.17	<0.0001	94.4%
*Vaccinated with 2 doses*	21	11,679,587	0.00180	(0.031–0.082)			(91.8–96.9)
**Age group**							
**18–59 years**							
Infection							
*Unvaccinated*	5903	11,550,681	0.51105	0.658	−25.28	<0.0001	**34.2%**
*Vaccinated with 2 doses*	9698	28,831,895	0.33636	(0.64–0.68)			(32.0–36.2)
Hospitalization							
*Unvaccinated*	254	11,178,574	0.02272	0.203	−14.47	<0.0001	**79.7%**
*Vaccinated with 2 doses*	122	26,418,502	0.00462	(0.16–0.25)			(74.8–83.6)
Death							
*Unvaccinated*	101	11,962,139	0.00844	0.044	−9.50	<0.0001	**95.6%**
*Vaccinated with 2 doses*	11	29,340,246	0.00037	(0.019–0.073)			(92.7–98.1)
**60 or more years**							
Infection							
*Unvaccinated*	401	823,716	0.48682	0.449	−13.30	<0.0001	**55.1%**
*Vaccinated with 2 doses*	862	3,943,266	0.21860	(0.40–0.50)			(49.6–60.3)
Hospitalization							
*Unvaccinated*	89	792,047	0.11237	0.121	−11.82	<0.0001	**87.9%**
*Vaccinated with 2 doses*	48	3,544,451	0.01354	(0.08–0.17)			(82.9–91.5)
Death							
*Unvaccinated*	104	832,546	0.12492	0.040	−13.01	<0.0001	**96.0%**
*Vaccinated with 2 doses*	20	3,988,793	0.00501	(0.022–0.060)			(94.0–97.8)
**Previous history of infection**							
**Absent**							
Infection							
*Unvaccinated*	5636	10,025,416	0.56217	0.716	−19.57	<0.0001	**28.4%**
*Vaccinated with 2 doses*	8923	22,156,861	0.40272	(0.693–0.741)			(25.9–30.7)
Hospitalization							
*Unvaccinated*	319	2,242,740	0.14224	0.113	−13.23	<0.0001	**88.7%**
*Vaccinated with 2 doses*	155	9,653,245	0.01606	(0.093–0.137)			(86.3–90.7)
Death							
*Unvaccinated*	191	10,398,828	0.01837	0.065	−13.19	<0.0001	**93.5%**
*Vaccinated with 2 doses*	27	22,621,886	0.00119	(0.040–0.092)			(90.8–96.0)
**Present**							
Infection							
*Unvaccinated*	668	2,348,981	0.28438	0.542	−13.32	<0.0001	**45.8%**
*Vaccinated with 2 doses*	1637	10,618,300	0.15417	(0.496–0.593)			(40.7–50.4)
Hospitalization							
*Unvaccinated*	24	2,242,740	0.01070	0.145	−5.86	<0.0001	**85.5%**
*Vaccinated with 2 doses*	15	9,653,245	0.00155	(0.076–0.277)			(72.3–92.4)
Death							
*Unvaccinated*	14	2,395,857	0.00584	0.032	−4.55	<0.0001	**96.8%**
*Vaccinated with 2 doses*	4	10,707,153	0.00037	(0.007–0.141)			(85.9–99.3)

* Updated 26 June 2021. ** There are significant differences (*p* < 0.0001) in the effectiveness of preventing infection, when comparing age groups and previous history of infection. There is no difference when comparing women and men in any of the 3 events.

## Data Availability

Not applicable.
